# O_2_ versus N_2_O respiration in a continuous microbial enrichment

**DOI:** 10.1007/s00253-018-9247-3

**Published:** 2018-07-27

**Authors:** Monica Conthe, Camiel Parchen, Gerben Stouten, Robbert Kleerebezem, Mark C. M. van Loosdrecht

**Affiliations:** Department of Biotechnology, Delft University of Technology, Van der Maasweg 9, 2629 HZ Delft, The Netherlands

**Keywords:** Nitrous oxide, Mixotrophy, Enrichment, Chemostat

## Abstract

Despite its ecological importance, essential aspects of microbial N_2_O reduction—such as the effect of O_2_ availability on the N_2_O sink capacity of a community—remain unclear. We studied N_2_O vs. aerobic respiration in a chemostat culture to explore (i) the extent to which simultaneous respiration of N_2_O and O_2_ can occur, (ii) the mechanism governing the competition for N_2_O and O_2_, and (iii) how the N_2_O-reducing capacity of a community is affected by dynamic oxic/anoxic shifts such as those that may occur during nitrogen removal in wastewater treatment systems. Despite its prolonged growth and enrichment with N_2_O as the sole electron acceptor, the culture readily switched to aerobic respiration upon exposure to O_2_. When supplied simultaneously, N_2_O reduction to N_2_ was only detected when the O_2_ concentration was limiting the respiration rate. The biomass yields per electron accepted during growth on N_2_O are in agreement with our current knowledge of electron transport chain biochemistry in model denitrifiers like *Paracoccus denitrificans*. The culture’s affinity constant (*K*_S_) for O_2_ was found to be two orders of magnitude lower than the value for N_2_O, explaining the preferential use of O_2_ over N_2_O under most environmentally relevant conditions.

**Electronic supplementary material:**

The online version of this article (10.1007/s00253-018-9247-3) contains supplementary material, which is available to authorized users.

## Introduction

Coping with rising levels of the potent greenhouse gas nitrous oxide (N_2_O) in the atmosphere calls for the development of mitigation strategies to reduce N_2_O accumulation and emission in soil management and wastewater treatment (WWT). The presence and activity of N_2_O-reducing organisms in fertilized soils and WWT plants, such as bacteria and archaea harboring *nosZ*-type genes, may be key in such mitigating strategies (Thomson et al. 2012). Nitrous oxide reductase (N_2_OR), the enzyme encoded by the *nosZ* gene, is a terminal reductase present in some microbial respiratory electron transport chains (ETC) that catalyzes the only microbial reaction known to consume N_2_O, converting it to innocuous N_2_ (which constitutes 79% of the Earth’s atmosphere). Although N_2_O reduction is generally associated to denitrifying organisms, many N_2_O reducers lack reductases other than N_2_OR (i.e., nitrate-, nitrite-, or nitric oxide-reductase; Hallin et al. 2018). However, most, if not all, denitrifiers—and presumably N_2_O reducers—are facultative aerobes, having the terminal oxidases necessary for O_2_ respiration (van Spanning and Richardson 2007).

Based on what is known on the biochemistry of model organisms like *Paracoccus denitrificans*, N_2_O and O_2_ respiration presumably share the core of the ETC (Chen and Strous 2013), with electrons branching out to O_2_ (via cytochrome oxidases), N_2_O (via N_2_OR), or other NO_x_ (in denitrifying N_2_O reducers) depending on electron acceptor availability. It is a common notion that, when both N_2_O and O_2_ are available, N_2_O reducers will consume O_2_ preferentially over N_2_O (and other N oxides; Shapleigh 2013). Even though N_2_O is a stronger electron acceptor than O_2_ in terms of thermodynamics, a number of authors have shown that N_2_O respiration is energetically less efficient than aerobic respiration, resulting in lower biomass growth yields per substrate (Koike and Hattori 1975; Stouthamer et al. 1982; Beun et al. 2000). We cannot rule out the existence of a more energy-efficient N_2_O reduction process (Conthe et al. 2018a), considering the broad phylogenetic diversity of N_2_O reducers and our limited knowledge regarding non-denitrifying N_2_O reducers in particular. However, given the growth yields reported in literature, it would make evolutionary sense for microorganisms to favor aerobic respiration over the respiration of N compounds to optimize energy conservation in the cell. Intriguingly, the physical mechanism directing electrons to O_2_ preferentially over other N compounds, when both electron acceptors are available, remains unclear.

Regulatory systems on a transcriptional or post-transcriptional level have been shown to shut down denitrification in the presence of oxygen in a variety of organisms (Zumft 1997). For instance, the NosZ protein of *Paracoccus denitrificans* and *Pseudomonas stutzeri* is inhibited by O_2_ in vitro (Coyle et al. 1985; Alefounder and Ferguson 1982), which could be a form of allosteric regulation in vivo. It has also been proposed that N_2_OR is—for reasons unknown—less competent than the cytochrome oxidases involved in respiration of O_2_ in the “competition” for electrons in the ETC (Qu et al. 2015). Nevertheless, diverse studies have reported the occurrence of denitrification in the presence of O_2_ (termed aerobic denitrification; Chen and Strous 2013 and references therein). Regarding N_2_O reduction more specifically, a significant degree of N_2_OR transcription and activity has been found under aerated conditions (Körner and Zumft 1989; Qu et al. 2015).

From a greenhouse gas mitigation point of view, it is interesting to study O_2_ and N_2_O mixotrophy—or the capability of microorganisms to simultaneously respire O_2_ and N_2_O—in order to understand how frequent oxic-anoxic shifts during nitrogen removal from wastewater, in space or time, may affect the N_2_O-reducing capacity of activated sludge. WWTP design and operation vary greatly, but universal questions to address are, e.g., (a) if N_2_OR activity can persist in aerated zones consuming nitrification-derived N_2_O potentially minimizing greenhouse gas emissions or (b) if, on the contrary, N_2_OR is relatively less active than the other NO_x_ reductases in the presence of O_2_, leading to N_2_O accumulation in the aerobic-anoxic transition zones.

We explored O_2_ versus N_2_O respiration in a continuous enrichment culture selected and grown with N_2_O as the sole electron acceptor and fully characterized—in terms of stoichiometry and community composition—in a previous study (Conthe et al. 2018b). The culture had been found to be composed of a relatively simple microbial community dominated by *Dechlorobacter*-like *Betaproteobacteria*. In this study, operation of the chemostat was continued and the N_2_O-limited steady-state conditions were intermittently interrupted to perform short-term batch experiments in situ, with varying concentrations of N_2_O, O_2_, or both N_2_O and O_2_ simultaneously, to determine (i) whether O_2_ is, in fact, preferentially consumed over N_2_O when both electron acceptors are available, (ii) under which O_2_ concentrations (if any) N_2_O consumption can take place, and (iii) to begin to unravel the mechanism governing the electron flow in the ETC to O_2_ or N_2_O.

## Materials and methods

### Chemostat operation

Following the work presented in Conthe et al. (2018b), a microbial enrichment using acetate as a carbon and energy source and exogenous N_2_O as the sole electron acceptor was maintained under N_2_O-limiting conditions in a continuous culture at 20 °C, pH 7, and a dilution rate of 0.026 ± 0.001 h^−1^. The reactor set-up, operation, sampling, and medium composition are described in detail in Conthe et al. (2018b, c). One hundred percent pure N_2_O gas diluted in Argon gas was fed to the chemostat at a total flow rate of 200 ml/min and the offgas from the reactor was recirculated at a rate of 700 ml/min, resulting in an incoming N_2_O concentration of roughly 0.30%. The stability of the culture in terms of conversion rates and microbial community composition was monitored by regular sampling of the broth and biomass and via online monitoring of the acid (1 M HCl) dosing (a proxy for acetate consumption in the system) and offgas composition.

### Batch experiments

The steady-state conditions of the culture were briefly interrupted on different operation days in order to perform batch experiments in situ and determine the maximum conversion rates of the enrichment under non-limiting conditions (Figure S1). The medium and effluent pumps were switched off and the gas supply rates of O_2_ (from a bottle of pure O_2_) and/or N_2_O were modified to achieve different electron acceptor concentrations within the system in random steps. Two main types of batches were performed: (1) supplying a single electron acceptor—either N_2_O or O_2_—at different concentrations or (2) supplying N_2_O and O_2_ simultaneously, keeping the N_2_O gas supply rate constant and varying that of O_2_. Additionally, we performed a batch test in which a constant O_2_ gas supply rate was maintained while varying that of N_2_O as well as short batch tests with either NO_3_^−^ or NO_2_^−^ to assess the denitrifying capacity of the culture. Note that gas recirculation was maintained during the experiments, causing an apparent delay between the conversions in the chemostat and the offgas concentration values measured. To avoid acetate depletion, a concentrated solution of sodium acetate was added to the broth at the start of the experiments and the 1 M HCl solution used for pH control during continuous operation was replaced by 1 M acetic acid for the duration of the experiment. For the batch tests with NO_3_^−^ and NO_2_^−^, these compounds were supplied as 1 M KNO_3_ or 1 M KNO_2_.

### Analytical procedures

Samples from the reactor for analysis of acetate and NH_4_^+^ were immediately filtered after sampling (0.45-μm pore size poly-vinylidene difluoride membrane, Merck Millipore, Carrigtohill, Ireland). Acetate was measured with a Chrompack CP 9001 gas chromatograph (Chrompack, Middelburg, The Netherlands) equipped with an HP Innowax column (Agilent Technologies, Santa Clara, CA, USA) and a flame ionization detector. Ammonium, NO_3_^−^, and NO_2_^−^ concentrations were determined spectrophotometrically using cuvette test kits (Hach Lange, Düsseldorf, Germany). For the estimation of biomass concentration, the volatile suspended solids (VSS) concentration was determined by centrifuging 0.2 L of the enrichment, drying the pellet overnight at 105 °C, and then burning the pellet at 550 °C for 2 h to determine the ash content. Additionally, the optical density of the culture (at a wavelength of 660; OD_660_) was monitored. Concentrations of N_2_O, N_2_ and CO_2_, Argon, and O_2_ in the headspace of the reactor were measured online via mass spectrometry (Prima BT, Thermo Scientific). The dissolved O_2_ concentration in the broth during the batch tests with O_2_ was measured with two types of oxygen sensors: a Clark electrode calibrated in the range of 0–20.8% and an optical oxygen probe calibrated in range 0–2% (Presens, Regensburg, Germany).

### Calculations

Elemental and electron balances during steady state were set up as described in Conthe et al. (2018a, b, c). During the batch tests, the conversion rates (r, in mol h^−1^) for O_2_ and N_2_O were calculated from the measured ingoing and outgoing gas composition and the argon supply rate (see Figures S2–S6 and Tables S2–S6 for details). The average biomass concentration value for each experimental step was derived from the ammonium uptake rates (see for example Figure S4b) and used to calculate the corresponding biomass specific rates (q, in mol CmolX^−1^ h^−1^). A standard and constant biomass composition of CH_1.8_O_0.5_N_0.2_ (Roels 1980). The qO_2_ and qN_2_O obtained for each step were plotted against the corresponding concentration of dissolved O_2_ or N_2_O in the broth in order to determine the *q*_max_ and *K*_s_ of the enrichment for O_2_ and N_2_O. The concentration of dissolved O_2_ was obtained experimentally with the DO probes while the concentration of dissolved N_2_O was estimated given a k_L_a_N2O_ of 180 h^−1^—obtained by scaling the experimentally derived *k*_L_a_O2_ (Janssen and Warmoeskerken 1987) and deriving the corresponding *K*_L_a_broth_ and *K*_L_a_headspace_ assuming a *t*_broth_ of 6 s (1800 and 50 h^−1^, respectively). A Monod model fitting the results was obtained by minimizing the sum of squared errors using the Microsoft Excel software.

The thermodynamic efficiency of metabolic growth using acetate as an electron donor and O_2_, N_2_O, or NO_3_^−^ as an electron acceptor can be interpreted by the Gibbs free energy (ΔG01) dissipated per C mole of biomass growth or per electron-equivalent used for respiration. These values were calculated based on Kleerebezem and van Loosdrecht (2010) and using the thermodynamic values found in Thauer et al. (1977)—please refer to Table S7 for more details.

### DNA extraction and 454 amplicon sequencing of 16S rRNA gene

The taxa-based community composition of the enriched culture during the period of operation presented in this study was determined by 454 amplicon sequencing of the 16S rRNA gene following the procedure described in Conthe et al. (2018a, b, c) and the sequences are available at NCBI under BioProject accession number PRJNA413885.

## Results

### Continuous operation and microbial community composition of the N_2_O-reducing enrichment

A culture enriched from activated sludge using acetate as a carbon source and electron donor and exogenous N_2_O as the sole electron acceptor was studied for a total period of 155 days (> 100 volume changes) in a chemostat under electron acceptor (N_2_O) limiting conditions (Figure S1). The start-up and characterization of the enrichment during the first 70 days of operation, in terms of conversion rates, stoichiometry, and microbial community composition, are described in Conthe et al. (2018b). During the subsequent period reported here, the conversion rates and corresponding biomass yields remained consistent with the previous period, characterized by steady-state growth on acetate oxidation coupled to N_2_O reduction to N_2_ (Tables 1 and 2). Furthermore, 454 amplicon sequencing of the 16S rRNA gene of the microbial community confirmed the continued prevalence of a *Dechlorobacter*-like OTU (Figure S1), transiently co-occurring (around day 100) with two other closely related OTUs classified as *Azonexus* and uncultured *Rhodocyclaceae*.

**Table 1:** Average biomass-specific conversion rates during steady state and the batch experiments

	Compound biomass specific conversion rates (mmol/mmol_X_ h^−1^)			
	*q*_N2O-N_	*q*_NO3-N_ or *q*_NO2-N_	*q*_*N2-N*_	*q*_Acetate- *C*_
Steady state	− 0.033 ± 0.001^b^		0.034 ± 0.001^b^	− 0.017 ± 0.001^b^
N_2_O batch	− 0.131 ± 0.004^b^		0.126 ± 0.008^b^	− 0.067 ± 0.009^c^
NO_3_^−^ batch		− 0.007 ± 0.000^c^	0.004 ± 0.000^c^	− 0.003 ± 0.000^c^
N_2_O + NO_2_^−^ batch^a^	− 0.033 ± 0.000^c^		0.042 ± 0.000^c^	

^a^N_2_O gas supply was kept on during addition of 1 mM KNO_2_^−^

^b^Standard deviation calculated from at least three independent measurements

^e^Standard deviation calculated by LINEST least squares method

**Table 2:** Experimentally determined biomass yields per mole of electron donor or per mole of electron equivalents respired during growth with N_2_O, NO_3_^−^, and O_2_ as an electron acceptor and corresponding Gibbs free energy dissipation values based on these yields

	Parameter	Units	Growth on electron acceptor		
			N_2_O^a^	NO_3_^-b^	O_2_^c^
Y_XS_	Biomass yield on acetate	Cmol_X_/Cmol_Ac-_	0.36 ± 0.03	0.38	0.45
Y_Xe_	Biomass yield on e^−^ transported in catabolic process	Cmol_X_/mol_e-_	0.16 ± 0.01	0.15	0.19
ΔG^01^_MET_	Metabolic energy change per mole donor^d^	kJ/Cmol_X_	− 1078	− 620	− 479
ΔG^01^_e CAT_	Metabolic energy change per electron transferred in catabolism	kJ/mol_e-_	− 159	− 96	− 101

^a^Steady state data, this study

^b^Steady state data—no siginificant accumulation of intermediates (Conthe et al.; data unpublished)

^c^Batch experiment data in N_2_O reducing enrichment, this study

### O_2_ vs. N_2_O batch tests: affinity and yields

**Fig. 1:**
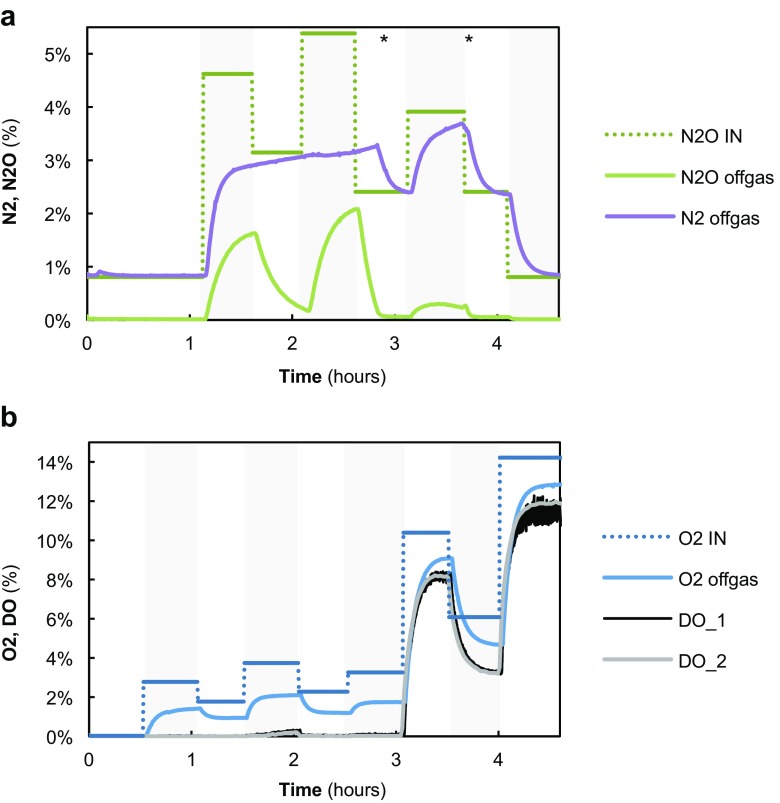
Offgas data from the batch experiments with varying concentrations of **a** N_2_O; day 106, **b** and O_2_; day 132. For the experiment with O_2_, the dissolved oxygen concentration (DO) was measured both with a Clark electrode (DO_1) and an optical sensor (DO_2). The affinity of the culture for N_2_O and O_2_ was determined from these experiments (see Fig. 5). The asterisk mark time points at which acetate had been depleted and was added to the culture

Batch experiments with varying supply rates of either N_2_O or O_2_ were performed on days 106 and 132, respectively (Fig. 1). The maximum biomass specific conversion rates of N_2_O (${q}_{\mathrm{N}2\mathrm{O}}^{\mathrm{max}}$) and acetate were identified by increasing the N_2_O supply rate to non-limiting conditions. The ${q}_{\mathrm{N}2\mathrm{O}}^{\mathrm{max}}$ values identified were roughly fourfold higher than the actual biomass specific conversion rates during steady state (Table 1). When exposed to varying concentrations of O_2_, the culture was able to switch to aerobic respiration in the order of seconds. The maximum O_2_ reducing capacity (${q}_{\mathrm{O}2}^{\mathrm{max}}$) was comparable to N_2_O respiration when expressed per mole electron accepted. NO_3_^−^ and NO_2_^−^ reducing capacities were much lower compared to N_2_O or O_2_ (< 15% of the maximum N_2_O or O_2_ reduction rate; Table 1).

**Fig. 2:**
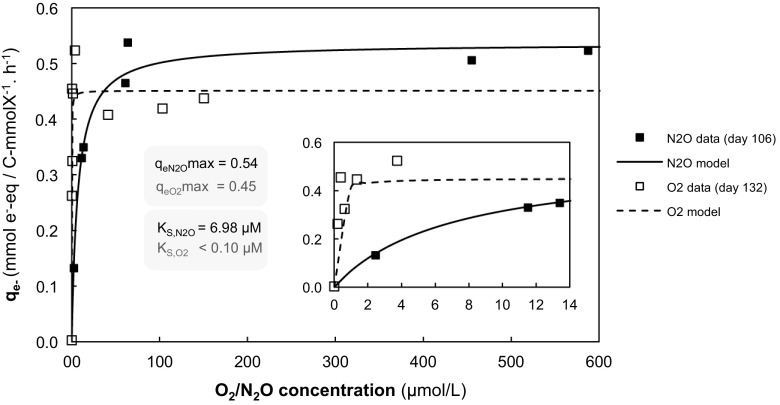
Biomass specific transfer rates of electron equivalents (*q*_e-_) as a function of the electron acceptor concentration (either N_2_O, in black, or O_2_, in gray), along with the fitting Monod model (with the corresponding *q*_e-_^max^ and *K*_s_ parameters). The inset is an enlargement of the graph at low O_2_/N_2_O concentrations. The rates presented were obtained from the experiments shown in Fig. 2

Plotting the biomass-specific electron transfer rate (*qe*^−^) at different dissolved O_2_ (DO) or N_2_O concentrations, we could determine the apparent *K*_s_ for O_2_ or N_2_O by fitting a Monod model to the data (Fig. 2). Given the confidence intervals, the absolute value for this parameter could not be identified accurately, but the results demonstrate clearly that the *K*_s_ value for O_2_ is 1 or 2 orders of magnitude smaller compared to *K*_s_-N_2_O. The maximum biomass-specific conversion rate of O_2_ (${q}_{\mathrm{O}2}^{\mathrm{max}}$) was roughly two times lower than that of N_2_O (${q}_{\mathrm{N}2\mathrm{O}}^{\mathrm{max}}$) per mole of electron acceptor but the conversion rates expressed as electron equivalents (${q}_{\mathrm{e}}^{\mathrm{max}}$) were comparable for both processes, since double the electrons are taken up during the reduction of O_2_ to H_2_O compared to N_2_O to N_2_.

The biomass yields per mole of electron donor (determined from the steady-state growth on N_2_O in the chemostat, and from the batch experiments with O_2_ as the sole electron acceptor) are presented in Table 2.

### Simultaneous O_2_ and N_2_O batch tests

**Fig. 3:**
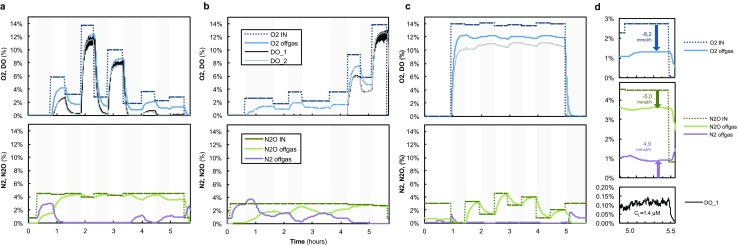
Offgas data from the batch experiments with excess N_2_O and varying concentrations of O_2_ on **a** day 125 and **b** day 155. The dissolved oxygen concentration (DO) was measured both with a Clark electrode (DO_1) and an optical sensor (DO_2). The biomass specific electron transfer rates to either N_2_O or O_2_ during these experiments are shown in Fig. 4. The asterisk marking the last two steps of the batch experiment on day 155 indicates the culture ran out of NH_4_^+^ for growth, and thus the rates during these steps was not considered. **c** Offgas data of batch experiment with excess O_2_ and varying concentrations of N_2_O on day 113. Detailed data from these experiments can be found in the Supplementary Materiasl—Tables xxx–xxx and Figures xxx to xxx. **d** Detailed view of one of the steps from the batch experiment depicted in (**a**) showing the simultaneous consumption of O_2_ and N_2_O, and subsequent production of N_2_

**Fig. 4:**
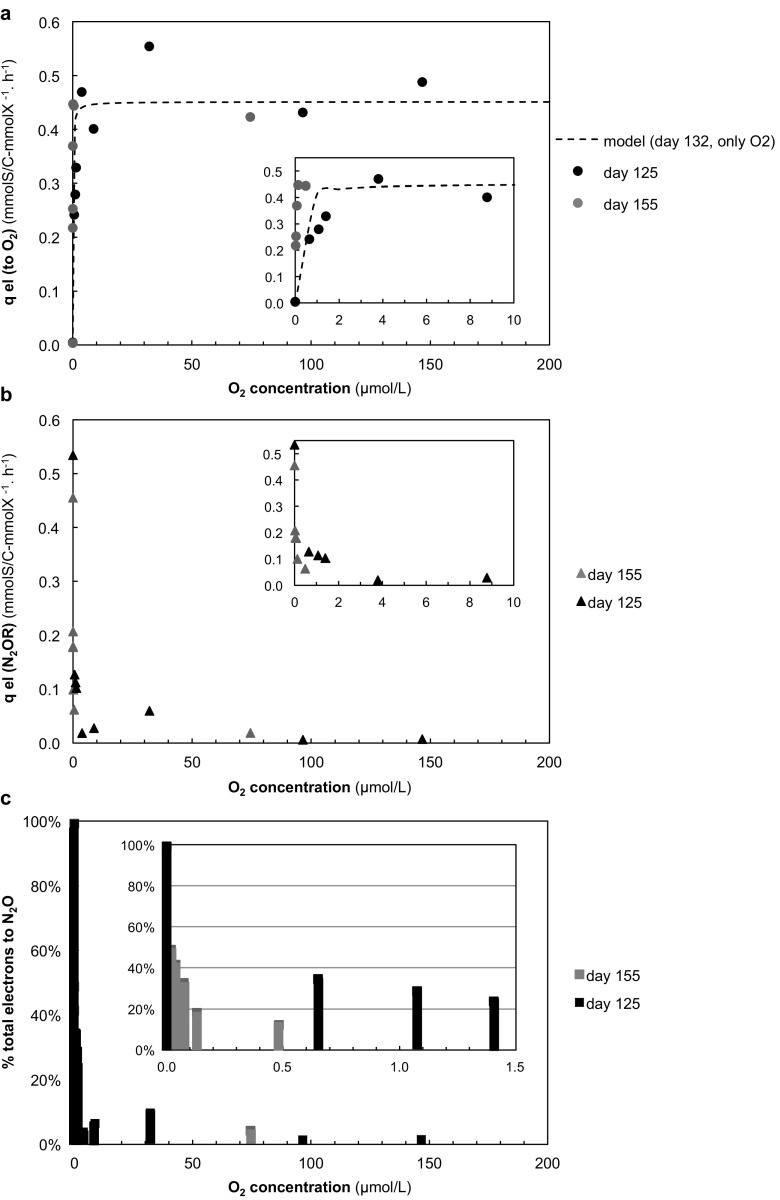
Biomass specific transfers rate of electron equivalents (*q*_e-_) (**a**) to O_2_ and **b** N_2_O and **c** percentage of total electrons being shuttled to N_2_O vs. O_2_ at varying O_2_ concentrations during the batch tests on day 125 (in black) and day 155 (in gray). The Monod model of O_2_ consumption in the absence of N_2_O (shown in Fig. 3) is included in (**a**) for comparison. The inset in (**c**) is an enlargement of the graph at low O_2_ concentrations

Batch experiments with excess N_2_O and varying concentrations of O_2_, supplied simultaneously, were performed on days 110 and 155 (Figs. 3 and 4). The maximum electron transfer rate (${q}_{\mathrm{e}}^{\mathrm{max}}$)—combining the electron transfer capacities of N_2_O and O_2_—summed up to a value comparable with the ${q}_{\mathrm{e}}^{\mathrm{max}}$ found during the N_2_O- or O_2_-only experiments. N_2_O reduction to N_2_ co-occurred with aerobic respiration only at relatively low concentrations of O_2_ (Fig. 3d). The experiments performed on days 110 and 155 differed regarding the O_2_ concentration range at which N_2_O reduction could co-occur (roughly < 4 and < 1.5 μM O_2_ on days 110 and 155, respectively) but, nevertheless, N_2_O reduction in the presence of O_2_ contributed to no more than a small fraction of the total electron acceptor capacity (generally < 20% of qe^—^tot; Fig. 4). An additional batch experiment on day 113, with a constant supply of O_2_ and a varying supply of N_2_O, also showed that N_2_O reduction was undetectable in the presence of relatively high concentrations of O_2_ (≈5 μM; Fig. 3c).

## Discussion

**Fig. 5:**
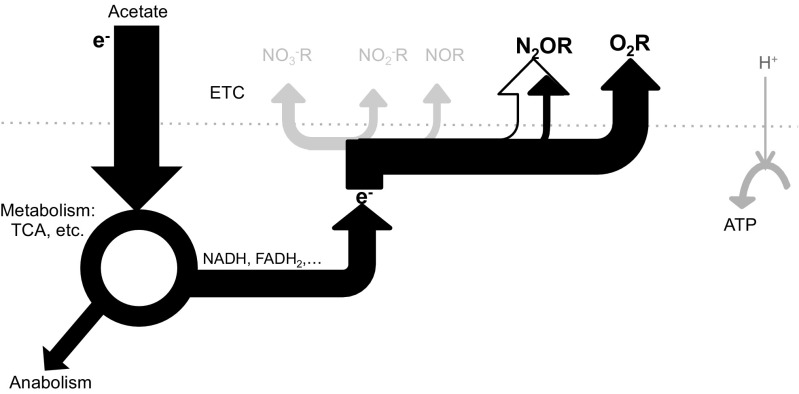
Simplified representation of the proportional distribution of electrons (e^−^) in the electron transport chain (ETC) during batch tests with only N_2_O (open arrow) versus batch tests with the simultaneous addition of O_2_ and N_2_O (black arrows) showing that there is a preferential shuttling of electrons to O_2_R than to N_2_OR. This simplified schematic is based on the assumptions that (i) both enzymes share a common electron pool (/quinone pool) and (ii) that all cells have a similar electron distribution among terminal reductases (whereas it would be possible for the majority of cells to switch fully to aerobic respiration, and a small fraction to continue respiring N_2_O)

Aerobic respiration was distinctly favored over N_2_O respiration in the enrichment despite the fact that the culture had been operated for an extensive number of generations with N_2_O as only electron acceptor. Upon a sudden change in supply from N_2_O to O_2_, the culture readily switched to O_2_ respiration and, when both electron acceptors were available, N_2_O reduction was only observed at relatively low concentrations of O_2_ (< 4 μM = 0.13 mg O_2_/L). Under conditions of electron acceptor excess (N_2_O and/or O_2_), growth in the system was likely limited by the electron supply rate to the electron transport chain (see Fig. 5) and not by the capacity of N_2_OR or O_2_ reductases. This was inferred from the fact that the maximum electron acceptor capacity of the culture was comparable for N_2_O and O_2_ respiration (i.e., ${q}_{{\mathrm{e}}^{-}\mathrm{N}2\mathrm{O}}^{\mathrm{max}}$ ≈ ${q}_{{\mathrm{e}}^{-}\mathrm{O}2}^{\mathrm{max}}$), and could be due to kinetic limitations in acetate uptake, acetate oxidation in the citric acid cycle, or in some shared component of the ETC itself.

The overall electron transfer capacity during the simultaneous respiration of N_2_O and O_2_ (i.e., ${q}_{{\mathrm{e}}^{-}\mathrm{TOT}}^{\mathrm{max}}$) was comparable to ${q}_{{\mathrm{e}}^{-}\mathrm{N}2\mathrm{O}}^{\mathrm{max}}$ or ${q}_{{\mathrm{e}}^{-}\mathrm{O}2}^{\mathrm{max}}$_2_. This suggests that “aerobic N_2_O respiration” (by analogy to aerobic denitrification) generally occurs if the electron supply rate to the ETC exceeds the electron accepting capacity of the O_2_ reductases. In other words, N_2_O respiration complements aerobic respiration primarily when O_2_ is limiting. Nonetheless, our results indicate that, under O_2_-limiting conditions, N_2_O reducers can use O_2_ and N_2_O mixotrophically as proposed by Chen and Strous 2013 (Fig. 5). We cannot exclude heterogeneity in electron acceptor use within the population in our bioreactor leading for example to most of the culture respiring O_2_ and a side population reducing N_2_O. Under the microscope, we did not observe formation of aggregates or biofilms which could create anoxic niches in spite of the O_2_ supply (data not shown), yet oxygen gradients and anoxic microzones could still form around suspended cells if O_2_ diffusion rate is slower than the respiration rate. Nevertheless, with the strong sparging and mixing conditions imposed on the culture, we would expect that most cells would be exposed to comparable environmental conditions.

The *K*_s_ values of the enrichment culture were in the same range as the *K*_m_ values reported for purified N_2_OR and different O_2_ reductases in literature, i.e., in the μM range for N_2_O and nM range for O_2_ (Pouvreau et al. 2008 and references therein, Yoon et al. 2016). The relatively high *K*_S,N2O_ (two orders of magnitude higher than for O_2_) is noteworthy in a culture presumably well-adapted to N_2_O-limiting conditions. Also the observation that, even after a prolonged absence of O_2_ in the environment, the cellular machinery specific for aerobic respiration (i.e., cytochrome oxidases) was constitutively present (in contrast to NO_3_^−^ and NO_2_^−^ reductases). According to these results, the preferential use of O_2_ over N_2_O in natural systems could be attributed to a difference in affinity (μmax/K_s_) for O_2_ and N_2_O.

With regard to efficiency of N_2_O respiration versus O_2_ respiration, our chemostat enrichment cultures corroborate studies in literature (Koike and Hattori 1975; Stouthamer et al. 1982; Beun et al. 2000) and predictions based on our knowledge of the ETC in model denitrifiers (Chen and Strous 2013): with biomass yields per mole of acetate during growth with N_2_O (or NO_3_) roughly 1/3 lower than yields during O_2_ respiration (Table 2). The relatively low growth yields on N_2_O imply that N_2_O reduction to N_2_ is, thermodynamically, a very inefficient process with high energy dissipation. Thus, ensuring the maximization of energy conservation during microbial growth may be the evolutionary driver behind the preferential flow of electrons to O_2_ over N_2_O.

We cannot provide a conclusive answer regarding which cellular mechanism governs the preferential use of O_2_ in the presence of excess N_2_O observed. However, the instantaneous switch from N_2_O to O_2_ respiration suggests that the preference for O_2_ over N_2_O is regulated at the metabolome level and is independent from transcriptional regulation, e.g., by control of enzyme activity, like allosteric inhibition of N_2_OR, or simply a higher affinity of O_2_ reductases for the electrons coming from a common quinone pool.

Translated to the environmental conditions in a WWT plant, the results from this study suggest that oxic-anoxic transitions are unlikely to result in N_2_O emissions associated to denitrification as a result of N_2_OR inhibition by O_2_ since the enrichment culture readily switched back and forth between O_2_ and N_2_O respiration. This implies that (a) either N_2_OR is not directly inhibited by O_2_ in vivo or (b) inhibition is readily reversible once O_2_ is depleted.

On the other hand, the fact that aerobic respiration is so strongly favored over N_2_O respiration would make it a challenge to exploit the N_2_O sink capacity of activated sludge in the aerated/nitrification zones of WWT plants. The range in which significant N_2_O consumption co-occurred with O_2_ consumption in our experiments was narrow: roughly up to 1.5–4 μM O_2_, i.e., 0.05–0.13 mg O_2_/L, presumably below common DO values in the aerated tanks of WWTP (Tchobanoglous and Burton 2002). The very high affinity for oxygen minimizes the range of dissolved oxygen concentrations in which O_2_ and N_2_O respiration could occur in parallel. However, a beneficial difference in full-scale systems compared to our enrichment, in terms of avoiding N_2_O accumulation, may be that mass transfer limitation induced oxygen limitation within the activated sludge flocs provide anoxic zones, prone to N_2_O reduction, even when O_2_ is present in the bulk liquid (Picioreanu et al. 2016). This, together with the fact that N_2_O is much more soluble than O_2_, could perhaps be exploited to enhance the N_2_O sink capacity of activated sludge.

## Electronic supplementary material

ESM 1 (PDF 912 kb)
