# Supplementary material for: O2 versus N2O respiration in a continuous microbial enrichment
Source: Appl Microbiol Biotechnol. 2018 Jul 27;102(20):8943–50. doi: 10.1007/s00253-018-9247-3 (PMC6153640; doi:10.1007/s00253-018-9247-3)
Supplement: Supplementary file 1 — (PDF 912 kb) [file 253_2018_9247_MOESM1_ESM.pdf]

Applied Microbiology and Biotechnology

O_2 versus N_2O respiration in a continuous microbial enrichment

Monica Conthe[1*], Camiel Parchen[1], Gerben Stouten[1], Robbert Kleerebezem[1], Mark C.M. van Loosdrecht[1]

[1]Department of Biotechnology, Delft University of Technology, Delft, The Netherlands

Correspondence:
Monica Conthe
Van der Maasweg 9, 2629 HZ Delft
Email: M.conthecalvo-24@tudelft.nl; Phone: +31 639 082 584

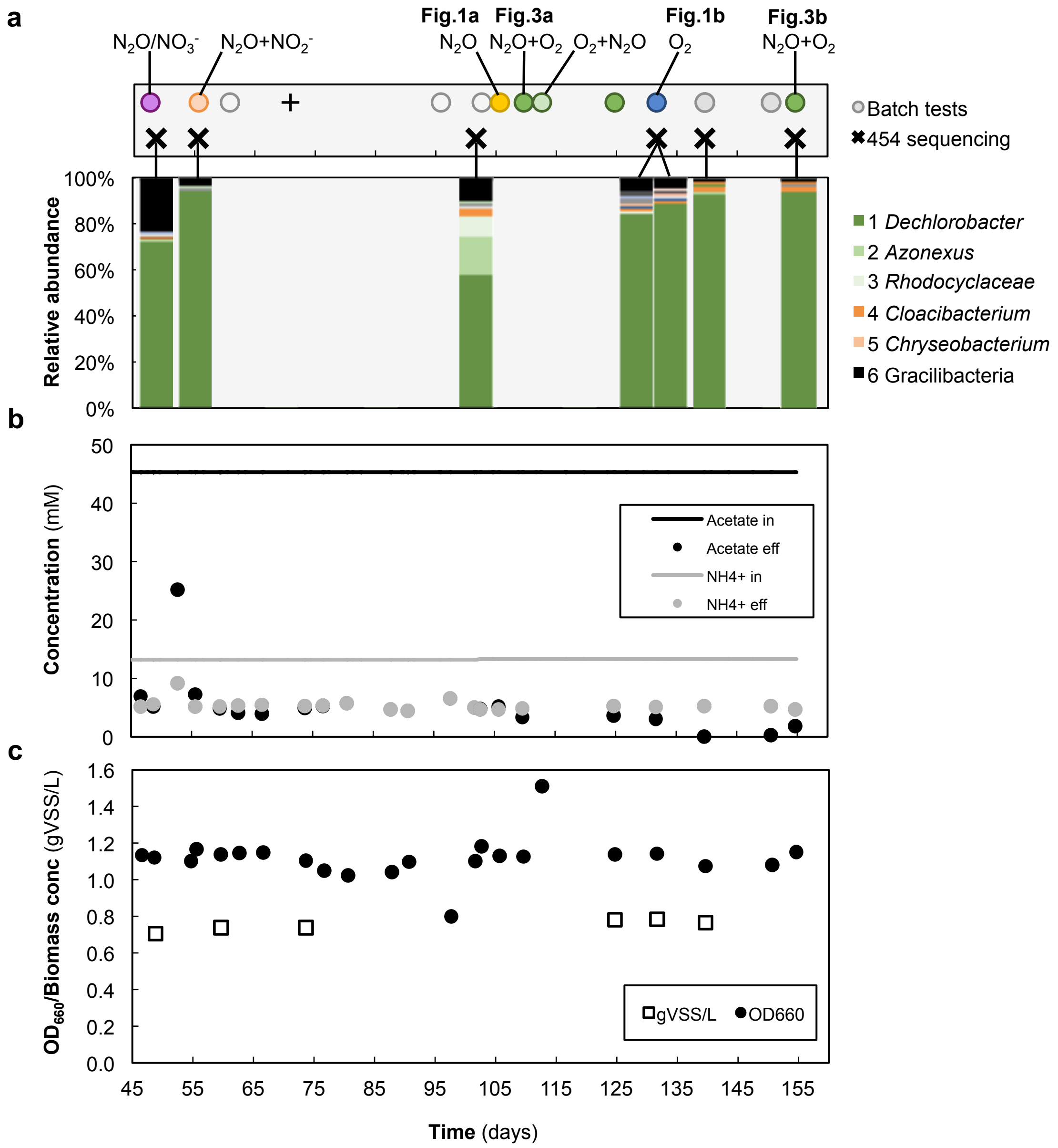

Figure S1 Chemostat operation under N_2O limitation (D = 0.026 h^{-1}, pH 7, 20°C) showing (**a**) the time points at which continuous operation was interrupted to perform batch experiments – marked with circles – together with the relative abundance of the main 16S rRNA gene OTUs making up the community, (**b**) incoming and outgoing acetate and NH_4^+ concentrations and, (**c**) biomass concentration (in gVSS/L) and optical density (OD_{660}) of the culture. The corresponding values prior to day 45 and during the start-up of the enrichment can be found in Conthe *et al.* (2018b). The biomass yields and biomass specific conversion rates are presented in Tables 1 and 2, in the main text, and the taxonomic assignment of 16S rRNA OTUs is available in Table S1.

Table S1 Assigned taxonomy for the main 16S rRNA sequences (i.e. those with > 5 % of total sequences in any given sample) using the Silva database

OTU	Kingdom	Phylum	Class	Order	Family	Genus	Identity (%) avg ± stdev
1	*Bacteria*	*Proteobacteria*	*Betaproteobacteria*	*Rhodocyclales*	*Rhodocyclaceae*	*Quatrionicoccus*	97.1 ± 0.6
2	*Bacteria*	*Proteobacteria*	*Betaproteobacteria*	*Rhodocyclales*	*Rhodocyclaceae*	uncultured	96.8 ± 0.9
3	*Bacteria*	*Proteobacteria*	*Betaproteobacteria*	*Rhodocyclales*	*Rhodocyclaceae*	*Azonexus*	97.6 ± 1.0
4	*Bacteria*	*Bacteroidetes*	*Flavobacteria*	*Flavobacteriales*	*Flavobacteriaceae*	*Cloacibacterium*	98.7 ± 0.8
5	*Bacteria*	*Bacteroidetes*	*Flavobacteria*	*Flavobacteriales*	Flavobacteriaceae	Chryseobacterium	97.9 ± 1.7
6	*Bacteria*	*Gracilibacteria*	---	---	---	---	84.8 ± 2.0

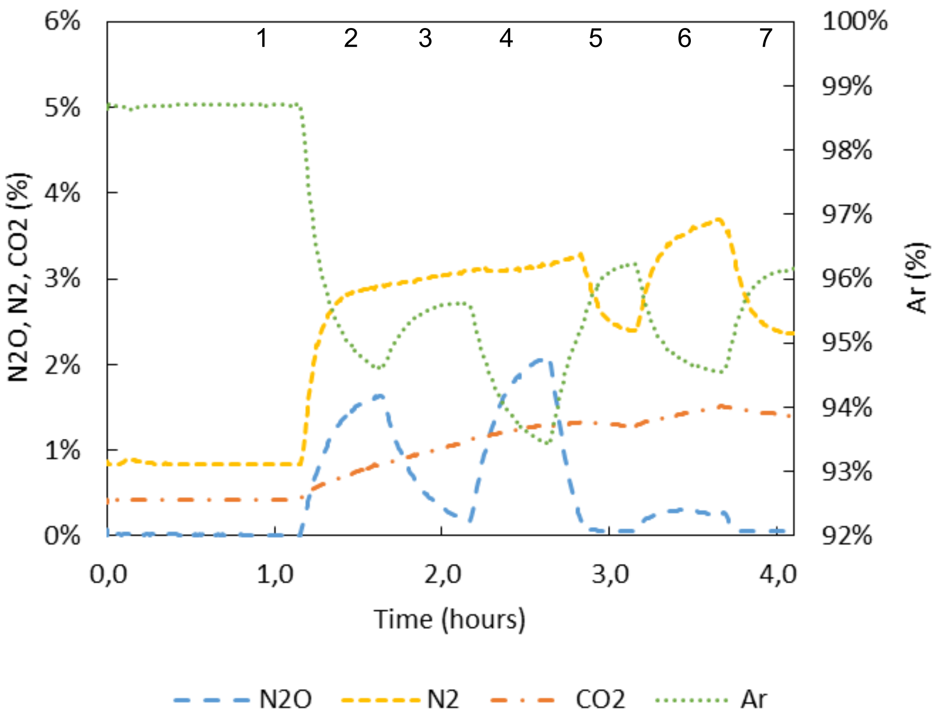

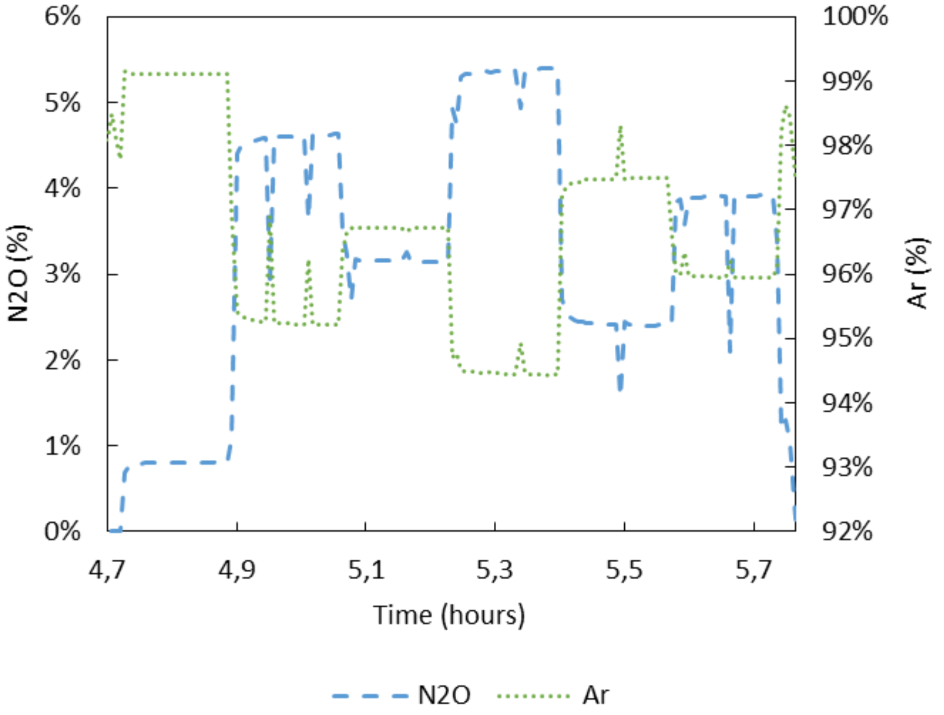

Figure S2 Concentration of N_2O, N_2, CO_2 and Argon in the offgas (above) and incoming gas (below) of the experiment on day 106 - N_2O only, presented in the main text as **Figure 1a**. The averaged data for each step – numbered in the graph - is presented in **Table S2**. Acetate was added manually to the culture during steps 5 and 7 to ensure that it was present in excess. NH_4^+ was also in excess throughout the experiment. pH was kept constant at 7.0 ± 0.1

Table S2 Average concentration and rates of N_2O, N_2, CO_2, O_2 and Argon supplied and produced during each of the steps (numbered 1 through 7) in the experiment with only N_2O on day 106 – presented in **Figure 1a** and **Figure S2.** Step 1 corresponds to steady state operation.

			IN-GAS *ml/min*	*mmol/h*		***OFF-gas*** *ml/min*	*mmol/h*	***R*** *mmol/h*	q_s *mol/(mol h)*	C_L *µM*
	N2	0,04%	0,07	0,20	0,83%	1,67	4,48	4,28	0,067	
	CO2	0,03%	0,05	0,14	0,43%	0,86	2,29	2,15	0,034	
**1**	N2O	0,81%	1,61	4,31	0,01%	0,03	0,07	-4,24	**-0,066**	**0,1**
	Ar	99,11%	197,97	530,27	98,70%	197,97	530,27	0	0,000	
	O2	0,02%	0,05	0,13	0,02%	0,05	0,13	0	0,000	
	N2	0,06%	0,13	0,35	2,89%	6,05	16,20	15,85	0,239	
	CO2	0,07%	0,14	0,38	0,81%	1,69	4,53	4,16	0,063	
**2**	N2O	4,62%	9,61	25,75	1,60%	3,34	8,96	-16,79	**-0,253**	**443,7**
	Ar	95,21%	197,97	530,27	94,67%	197,97	530,27	0	0,000	
	O2	0,03%	0,07	0,18	0,03%	0,06	0,17	-0,01	0,000	
	N2	0,05%	0,11	0,28	3,06%	6,35	17,00	16,71	0,243	
	CO2	0,05%	0,10	0,26	1,07%	2,22	5,94	5,67	0,083	
**3**	N2O	3,15%	6,44	17,25	0,23%	0,48	1,27	-15,98	**-0,232**	**50,2**
	Ar	96,72%	197,97	530,27	95,61%	197,97	530,27	0	0,000	
	O2	0,03%	0,06	0,16	0,03%	0,05	0,14	-0,02	0,000	
	N2	0,07%	0,14	0,37	3,15%	6,68	17,89	17,52	0,247	
	CO2	0,07%	0,15	0,41	1,28%	2,72	7,29	6,88	0,097	
**4**	N2O	5,38%	11,29	30,23	2,06%	4,37	11,70	-18,54	**-0,261**	**574,1**
	Ar	94,44%	197,97	530,27	93,47%	197,97	530,27	0	0,000	
	O2	0,03%	0,07	0,19	0,03%	0,06	0,15	-0,04	-0,001	
	N2	0,05%	0,09	0,25	2,41%	4,96	13,30	13,05	0,179	
	CO2	0,03%	0,07	0,18	1,29%	2,65	7,10	6,91	0,095	
**5**	N2O	2,41%	4,89	13,09	0,06%	0,13	0,33	-12,76	**-0,175**	**4,3**
	Ar	97,49%	197,97	530,27	96,21%	197,97	530,27	0	0,000	
	O2	0,03%	0,06	0,15	0,03%	0,06	0,15	0,00	0,000	
	N2	0,05%	0,11	0,30	3,67%	7,69	20,59	20,30	0,269	
	CO2	0,05%	0,11	0,30	1,49%	3,13	8,38	8,08	0,107	
**6**	N2O	3,91%	8,07	21,62	0,24%	0,51	1,37	-20,25	**-0,269**	**48,1**
	Ar	95,95%	197,97	530,27	94,56%	197,97	530,27	0	0,000	
	O2	0,03%	0,06	0,16	0,03%	0,06	0,15	-0,01	0,000	
	N2	0,05%	0,09	0,25	2,37%	4,89	13,09	12,84	0,166	
	CO2	0,03%	0,07	0,18	1,41%	2,90	7,76	7,58	0,098	
**7**	N2O	2,41%	4,89	13,09	0,05%	0,11	0,30	-12,80	**-0,165**	**2,1**
	Ar	97,49%	197,97	530,27	96,14%	197,97	530,27	0	0,000	
	O2	0,03%	0,06	0,15	0,03%	0,05	0,14	-0,01	0,000	

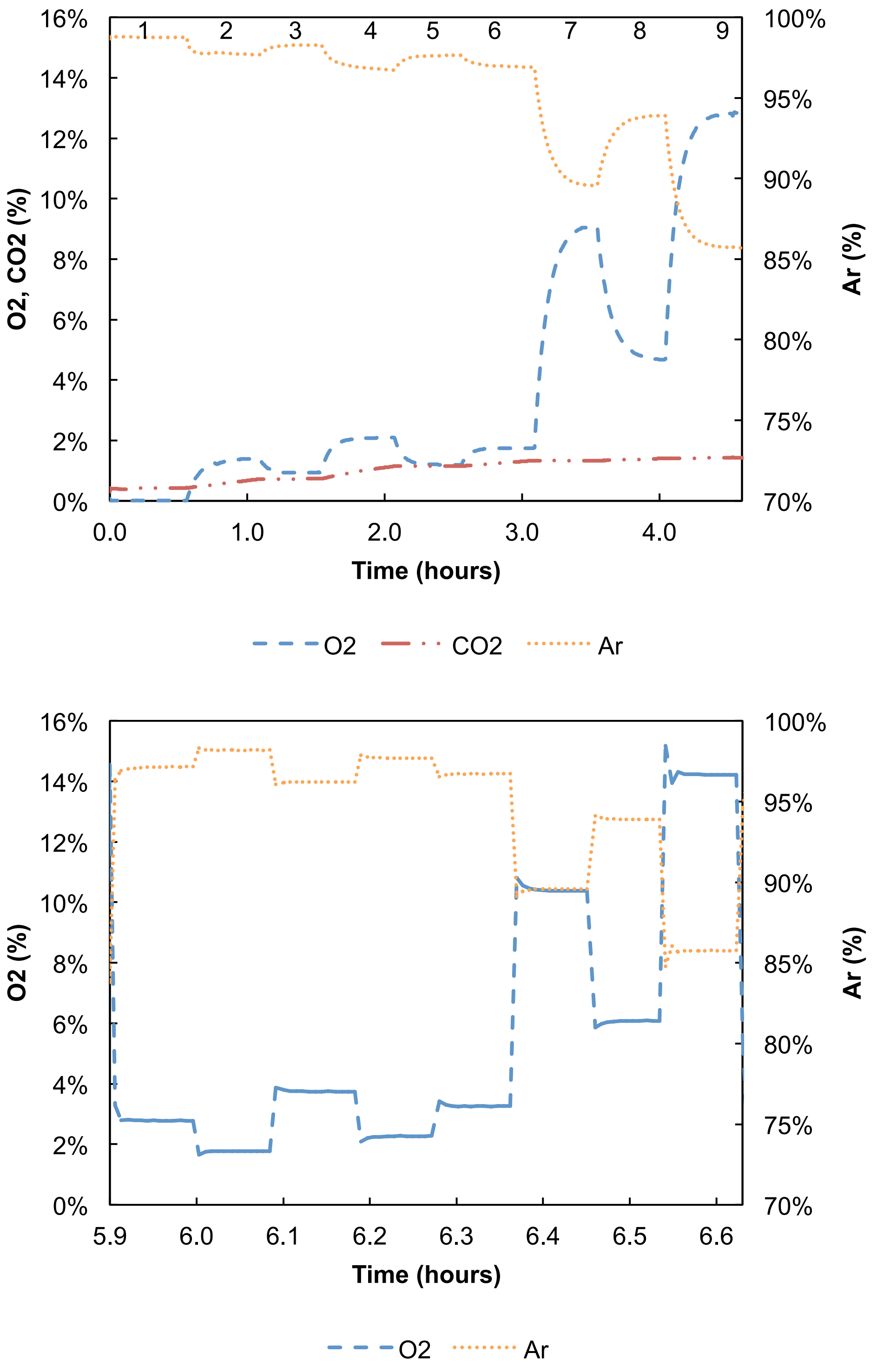

Figure S3 Concentration of N_2O, N_2, CO_2 and Argon in the offgas (above) as well as the incoming gas (below) of the experiment on day 132 (O_2 only) – presented in the main text as **Figure 1b**. The averaged data for each step – numbered in the graph - is presented in **Table S3**. Acetate and NH_4^+ were present in excess throughout the experiment. pH was kept constant at 7.0 ± 0.1

Table S3 Average concentration and rates of N_2O, N_2, CO_2, O_2 and Argon supplied and produced during each of the steps (numbered 1 through 9) in the experiment with only O_2 on day 132 – **Figure 1b** in the main text and **Figure S3**. Step 1 corresponds to steady state operation.

			IN-GAS *ml/min*	*mmol/h*		***OFF-gas*** *ml/min*	*mmol/h*	***R*** *mmol/h*	***q_s*** *mol/(mol h)*	***C_L*** *μM*
	N2	0,03%	0,06	0,17	0,80%	1,60	4,28	4,11	0,064	
	CO2	0,02%	0,05	0,13	0,43%	0,85	2,29	2,15	0,034	
**1**	N2O	0,77%	1,54	4,13	0,01%	0,02	0,04	-4,08	-0,064	
	Ar	99,15%	197,97	530,27	98,75%	197,97	530,27	0,00	0,000	
	O2	0,02%	0,05	0,12	0,01%	0,03	0,08	-0,05	**-0,001**	**0,00**
	N2	0,02%	0,04	0,10	0,21%	0,42	1,13	1,03	0,016	
	CO2	0,01%	0,02	0,06	0,69%	1,40	3,75	3,69	0,056	
**2**	N2O	0,03%	0,06	0,17	0,00%	0,00	0,00	-0,17	-0,003	
	Ar	97,16%	197,97	530,27	97,70%	197,97	530,27	0	0,000	
	O2	2,78%	5,66	15,15	1,40%	2,84	7,60	-7,55	**-0,114**	**0,39**
	N2	0,02%	0,04	0,10	0,06%	0,11	0,30	0,21	0,003	
	CO2	0,01%	0,02	0,05	0,74%	1,48	3,97	3,92	0,057	
**3**	N2O	0,01%	0,03	0,08	0,00%	0,00	0,00	-0,08	-0,001	
	Ar	98,18%	197,97	530,27	98,27%	197,97	530,27	0	0,000	
	O2	1,77%	3,58	9,59	0,94%	1,89	5,07	-4,52	**-0,066**	**0,20**
	N2	0,02%	0,04	0,10	0,04%	0,07	0,20	0,10	0,001	
	CO2	0,01%	0,02	0,04	1,11%	2,28	6,10	6,06	0,087	
**4**	N2O	0,01%	0,02	0,05	0,00%	0,00	0,00	-0,05	-0,001	
	Ar	96,22%	197,97	530,27	96,75%	197,97	530,27	0	0,000	
	O2	3,74%	7,70	20,61	2,10%	4,29	11,50	-9,12	**-0,131**	**3,72**
	N2	0,02%	0,04	0,10	0,03%	0,06	0,15	0,05	0,001	
	CO2	0,01%	0,02	0,04	1,15%	2,33	6,23	6,19	0,086	
**5**	N2O	0,00%	0,01	0,02	0,00%	0,00	0,00	-0,02	0,000	
	Ar	97,70%	197,97	530,27	97,63%	197,97	530,27	0	0,000	
	O2	2,27%	4,60	12,33	1,19%	2,42	6,48	-5,85	**-0,081**	**0,62**
	N2	0,02%	0,04	0,10	0,03%	0,06	0,16	0,06	0,001	
	CO2	0,01%	0,01	0,04	1,31%	2,68	7,18	7,14	0,096	
**6**	N2O	0,00%	0,00	0,01	0,00%	0,00	0,00	-0,01	0,000	
	Ar	96,71%	197,97	530,27	96,91%	197,97	530,27	0	0,000	
	O2	3,26%	6,67	17,87	1,75%	3,57	9,56	-8,31	**-0,112**	**1,35**
	N2	0,02%	0,04	0,11	0,02%	0,05	0,14	0,02	0,000	
	CO2	0,01%	0,02	0,04	1,33%	2,93	7,84	7,80	0,104	
**7**	N2O	0,00%	0,00	0,00	0,00%	0,00	0,00	0,00	0,000	
	Ar	89,58%	197,97	530,27	89,59%	197,97	530,27	0	0,000	
	O2	10,39%	22,96	61,49	9,06%	20,02	53,64	-7,86	**-0,105**	**103,4**
	N2	0,02%	0,04	0,10	0,02%	0,05	0,14	0,04	0,001	
	CO2	0,01%	0,01	0,04	1,40%	2,94	7,89	7,85	0,101	
**8**	N2O	0,00%	0,00	0,00	0,00%	0,00	0,00	0,00	0,000	
	Ar	93,89%	197,97	530,27	93,90%	197,97	530,27	0	0,000	
	O2	6,08%	12,82	34,35	4,68%	9,87	26,45	-7,90	**-0,102**	**41,4**
	N2	0,02%	0,04	0,10	0,02%	0,05	0,14	0,04	0,000	
	CO2	0,01%	0,01	0,04	1,43%	3,30	8,83	8,79	0,110	
**9**	N2O	0,00%	0,00	0,01	0,00%	0,00	0,00	-0,01	0,000	
	Ar	85,76%	197,97	530,27	85,74%	197,97	530,27	0	0,000	
	O2	14,22%	32,82	87,91	12,80%	29,56	79,19	-8,72	**-0,109**	**150,5**

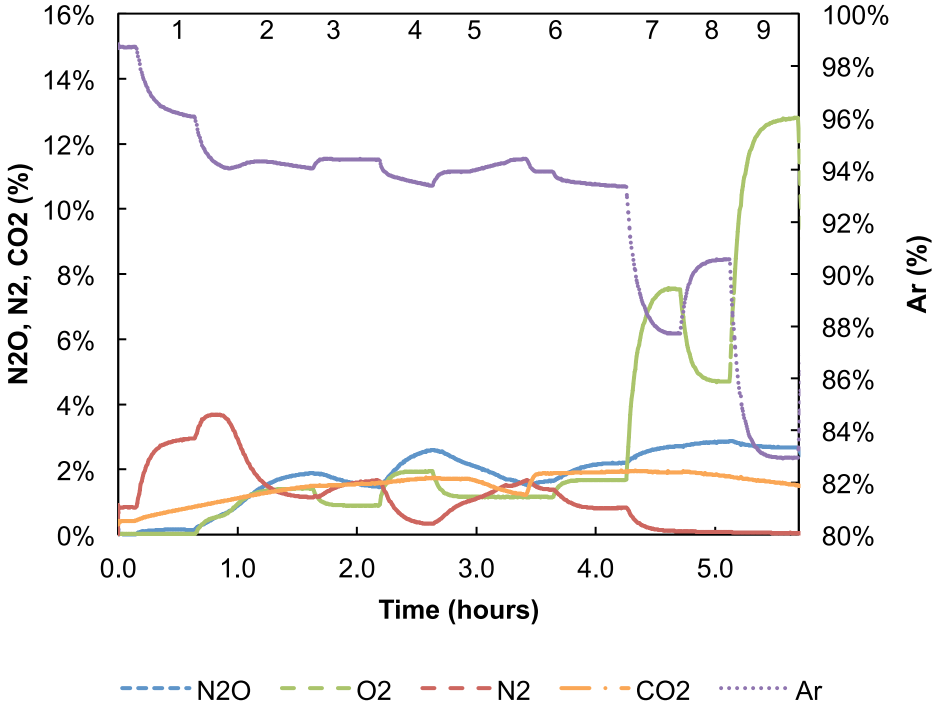

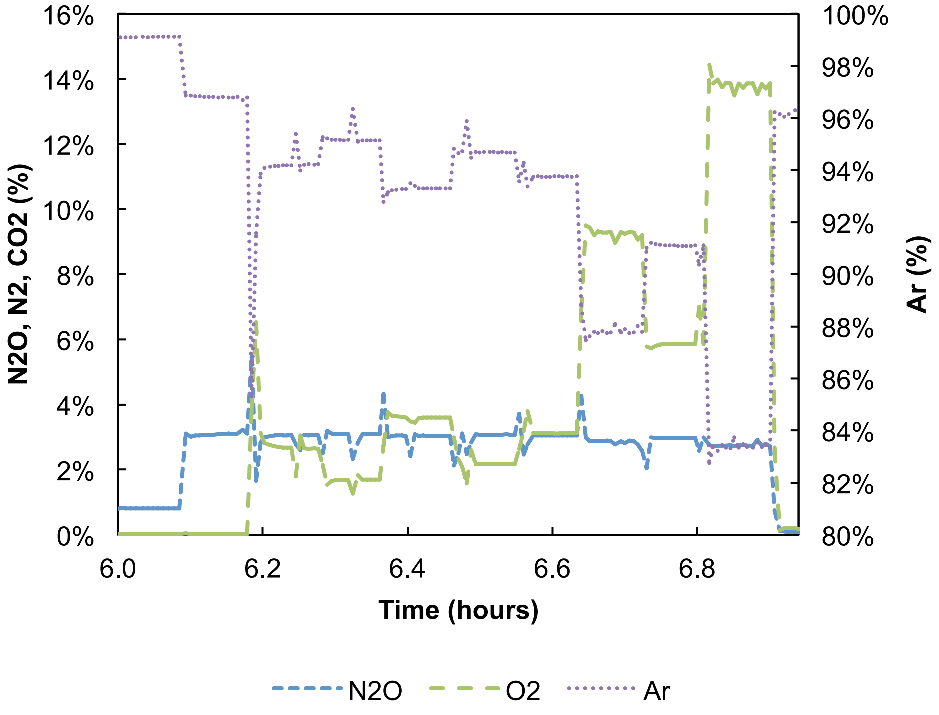

Figure S4 Concentration N_2O, N_2, CO_2, O_2 and Argon in the offgas (above) and incoming gas (below) of the experiment on day 110 (N_2O + O_2) – **Figure 3a** in the main text. The averaged data for each step – numbered in the graph - is presented in **Table S4**. Acetate and NH_4^+ were present in excess throughout the experiment. pH was kept constant at 7.0 ± 0.1

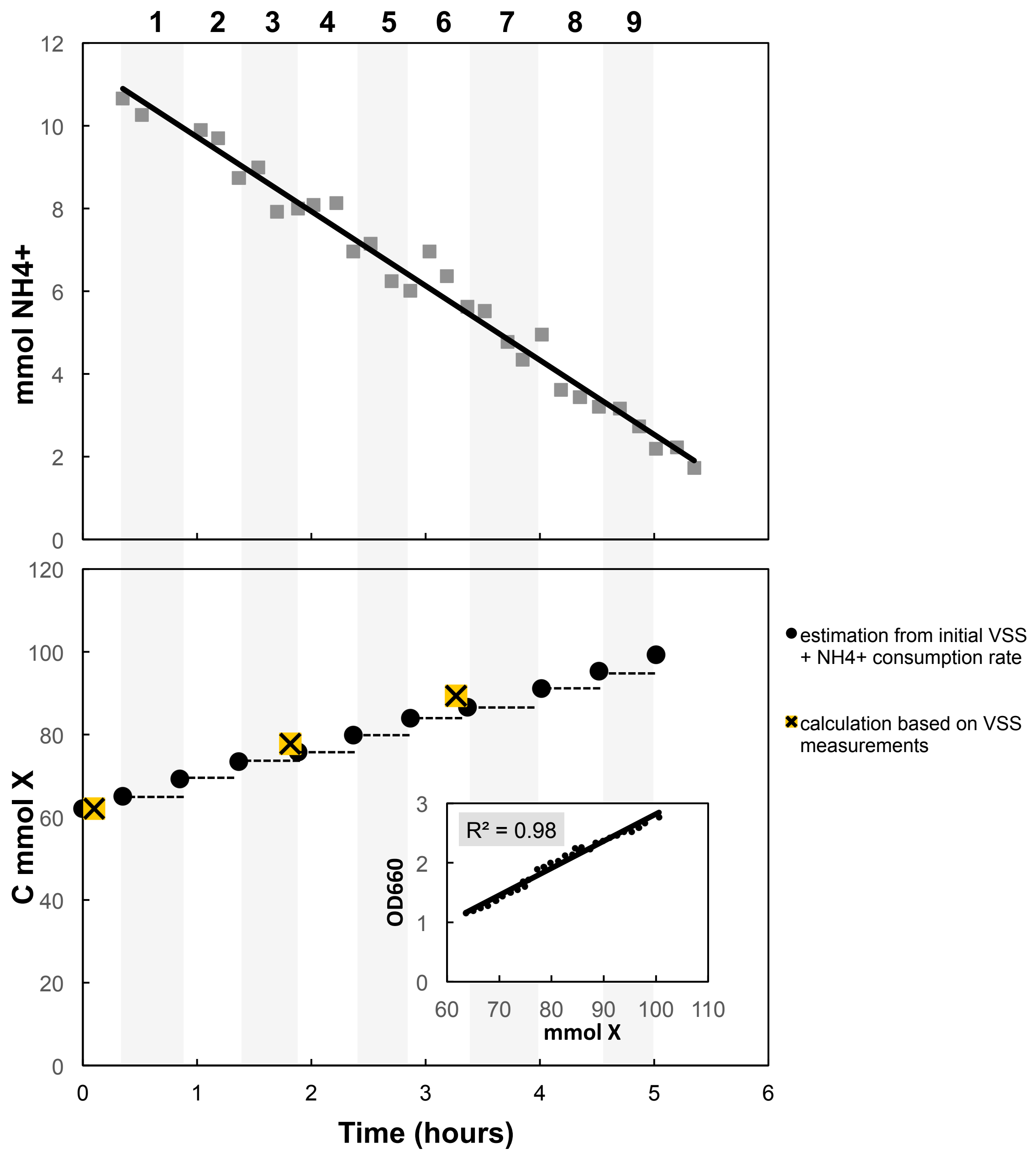

Figure S4b Amount of NH_4^+ (above) and biomass (below) during the 9 steps of the experiment corrected for the broth volume in the chemostat. The average biomass valies for each step was calculated based on the initial VSS measurement (assuming a molar weight of 24,6 g/mol) corrected for biomass growth during each step. This growth was estimated based on the NH_4^+ consumption shown above, assuming biomass contains 0,2 N - mole per C-mole. This estimation correlates with the VSS measurements performed at the end of steps 3 and 6 and with the OD_{660}, as shown in the inset. These values were used to calculate the biomass specific rates (q) for each step.

Table S4 Average concentration and rates of N_2O, N_2, CO_2, O_2 and Argon supplied and produced during each of the steps (numbered 1 through 9) in the experiment with simultaneous presence of O_2 and N_2O on day 110 – **Figure 3a** in the main text and **Figure S4**.

			IN-GAS *ml/min*	*mmol/h*		***OFF-gas*** *ml/min*	*mmol/h*	***R*** *mmol/h*	***q_s*** *mol/(mol h)*	***C_L*** *µM*
	N2	0,04%	0,08	0,21	2,94%	6,06	16,23	16,02	0,226	
	CO2	0,04%	0,08	0,22	0,82%	1,68	4,51	4,29	0,061	
**1**	N2O	3,09%	6,33	16,95	0,15%	0,30	0,82	-16,13	-0,228	
	Ar	96,80%	197,97	530,27	96,08%	197,97	530,27	0,00	0,000	
	O2	0,03%	0,06	0,16	0,02%	0,03	0,09	-0,06	-0,001	**0,00**
	N2	0,04%	0,08	0,22	1,15%	2,43	6,50	6,27	0,084	
	CO2	0,04%	0,08	0,20	1,48%	3,11	8,33	8,12	0,108	
**2**	N2O	3,06%	6,43	17,23	1,87%	3,94	10,55	-6,68	-0,089	
	Ar	94,22%	197,97	530,27	94,08%	197,97	530,27	0	0,000	
	O2	2,65%	5,56	14,89	1,41%	2,98	7,97	-6,92	-0,092	**0,07**
	N2	0,04%	0,08	0,21	1,65%	3,47	9,28	9,08	0,109	
	CO2	0,07%	0,14	0,38	1,57%	3,29	8,81	8,43	0,101	
**3**	N2O	3,04%	6,33	16,97	1,49%	3,12	8,35	-8,62	**-0,104**	
	Ar	95,14%	197,97	530,27	94,40%	197,97	530,27	0	0,000	
	O2	1,71%	3,57	9,55	0,90%	1,88	5,03	-4,52	**-0,054**	**0,02**
	N2	0,04%	0,08	0,23	0,34%	0,71	1,91	1,69	0,020	
	CO2	0,04%	0,08	0,20	1,74%	3,68	9,86	9,66	0,114	
**4**	N2O	3,04%	6,44	17,26	2,58%	5,46	14,62	-2,64	**-0,031**	
	Ar	93,29%	197,97	530,27	93,41%	197,97	530,27	0	0,000	
	O2	3,60%	7,63	20,44	1,94%	4,11	11,00	-9,44	**-0,111**	**0,48**
	N2	0,04%	0,08	0,22	1,39%	2,93	7,86	7,63	0,086	
	CO2	0,04%	0,07	0,20	1,88%	3,96	10,62	10,42	0,117	
**5**	N2O	3,08%	6,43	17,23	1,64%	3,45	9,24	-7,99	**-0,090**	
	Ar	94,68%	197,97	530,27	93,94%	197,97	530,27	0	0,000	
	O2	2,17%	4,53	12,14	1,15%	2,43	6,51	-5,63	**-0,063**	**0,04**
	N2	0,04%	0,08	0,23	0,82%	1,74	4,67	4,45	0,046	
	CO2	0,04%	0,08	0,21	1,94%	4,10	10,99	10,78	0,110	
**6**	N2O	3,05%	6,47	17,32	2,20%	4,66	12,48	-4,85	**-0,050**	
	Ar	93,29%	197,97	530,27	93,37%	197,97	530,27	0	0,000	
	O2	3,60%	7,63	20,44	1,68%	3,55	9,52	-10,92	**-0,112**	**0,13**
	N2	0,04%	0,09	0,25	0,11%	0,25	0,66	0,41	0,004	
	CO2	0,02%	0,05	0,13	1,91%	4,32	11,57	11,44	0,115	
**7**	N2O	2,87%	6,47	17,33	2,71%	6,12	16,40	-0,93	**-0,009**	
	Ar	87,78%	197,97	530,27	87,72%	197,97	530,27	0	0,000	
	O2	9,28%	20,94	56,08	7,54%	17,02	45,58	-10,50	**-0,106**	**74,5**
	N2	0,04%	0,09	0,23	0,08%	0,17	0,46	0,23	0,002	
	CO2	0,03%	0,07	0,19	1,80%	3,93	10,53	10,34	0,103	
**8**	N2O	2,97%	6,45	17,28	2,86%	6,25	16,73	-0,55	**-0,005**	
	Ar	91,10%	197,97	530,27	90,57%	197,97	530,27	0	0,000	
	O2	5,86%	12,74	34,11	4,70%	10,27	27,50	-6,61	**-0,066**	**46,6**
	N2	0,04%	0,09	0,24	0,05%	0,12	0,33	0,09	0,001	
	CO2	0,02%	0,04	0,12	1,56%	3,72	9,95	9,84	0,098	
**9**	N2O	2,75%	6,52	17,48	2,68%	6,39	17,13	-0,35	**-0,003**	
	Ar	83,39%	197,97	530,27	82,95%	197,97	530,27	0	0,000	
	O2	13,81%	32,79	87,82	12,76%	30,45	81,57	-6,25	**-0,062**	**160,0**

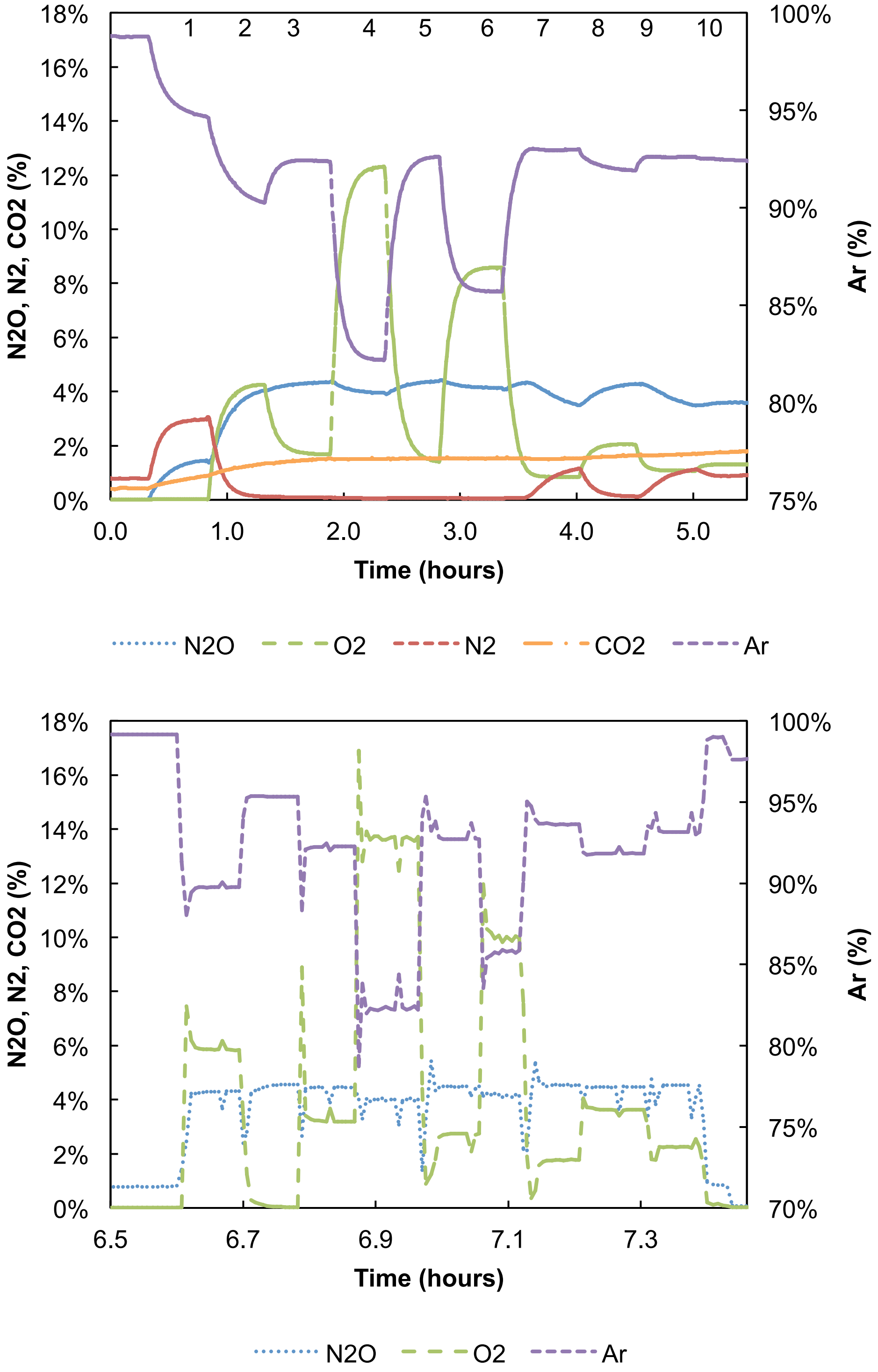

Figure S5 Concentration of N_2O, N_2, CO_2, O_2 and Argon in the offgas (above) and incoming gas (below) of the experiment on day 155 (N_2O + O_2) – **Figure 3b** in the main text. The averaged data for each step is presented in **Table S5**. Acetate and NH_4^+ were present in excess throughout the experiment. pH was kept constant at 7.0 ± 0.1

Table S5 Average concentration and rates of N_2O, N_2, CO_2, O_2 and Argon supplied and produced during each of the steps (numbered 1 through 10) in the experiment with simultaneous presence of O_2 and N_2O on day 155- **Figure 3b** in the main text and **Figure S5**.

			IN-GAS			***OFF-gas***		***R***	q_s	C_L
			ml/min	*mmol/h*		*ml/min*	*mmol/h*	*mmol/h*	*mol/(mol h)*	*µM*
	N2	0,05%	0,10	0,26	2,96%	6,18	16,55	16,29	0,251	
	CO2	0,04%	0,08	0,23	0,84%	1,75	4,70	4,47	0,069	
**1**	N2O	4,56%	9,46	25,34	1,43%	2,98	7,98	-17,36	**-0,267**	
	Ar	95,33%	197,97	530,27	94,76%	197,97	530,27	0,00	0,000	
	O2	0,03%	0,06	0,17	0,02%	0,03	0,09	-0,08	**-0,001**	**0,00**
	N2	0,05%	0,11	0,29	0,13%	0,28	0,75	0,45	0,007	
	CO2	0,04%	0,09	0,23	1,31%	2,86	7,67	7,45	0,108	
**2**	N2O	4,31%	9,51	25,47	3,99%	8,73	23,40	-2,07	**-0,030**	
	Ar	89,76%	197,97	530,27	90,33%	197,97	530,27	0	0,000	
	O2	5,84%	12,89	34,52	4,25%	9,31	24,93	-9,59	**-0,139**	**32,1**
	N2	0,05%	0,10	0,27	0,08%	0,18	0,47	0,20	0,003	
	CO2	0,04%	0,08	0,22	1,51%	3,24	8,69	8,47	0,115	
**3**	N2O	4,46%	9,56	25,61	4,34%	9,31	24,93	-0,68	**-0,009**	
	Ar	92,27%	197,97	530,27	92,38%	197,97	530,27	0	0,000	
	O2	3,18%	6,83	18,28	1,68%	3,61	9,66	-8,63	**-0,117**	**3,79**
	N2	0,05%	0,11	0,30	0,06%	0,13	0,36	0,06	0,001	
	CO2	0,04%	0,09	0,24	1,51%	3,63	9,71	9,47	0,125	
**4**	N2O	4,01%	9,65	25,84	3,96%	9,54	25,56	-0,28	**-0,004**	
	Ar	82,19%	197,97	530,27	82,19%	197,97	530,27	0	0,000	
	O2	13,72%	33,04	88,49	12,28%	29,58	79,24	-9,25	**-0,122**	**146,6**
	N2	0,05%	0,10	0,27	0,06%	0,14	0,37	0,10	0,001	
	CO2	0,03%	0,06	0,16	1,53%	3,28	8,78	8,62	0,108	
**5**	N2O	4,49%	9,58	25,66	4,37%	9,35	25,04	-0,62	**-0,008**	
	Ar	92,70%	197,97	530,27	92,58%	197,97	530,27	0	0,000	
	O2	2,74%	5,85	15,66	1,45%	3,09	8,29	-7,37	**-0,092**	**2,55**
	N2	0,04%	0,10	0,27	0,05%	0,12	0,33	0,06	0,001	
	CO2	0,02%	0,05	0,14	1,53%	3,52	9,44	9,30	0,111	
**6**	N2O	4,18%	9,66	25,88	4,14%	9,57	25,63	-0,25	**-0,003**	
	Ar	85,71%	197,97	530,27	85,70%	197,97	530,27	0	0,000	
	O2	10,04%	23,19	62,11	8,57%	19,81	53,05	-9,05	**-0,108**	**96,6**
	N2	0,05%	0,10	0,26	1,11%	2,37	6,35	6,10	0,070	
	CO2	0,02%	0,05	0,14	1,53%	3,25	8,71	8,57	0,099	
**7**	N2O	4,54%	9,59	25,70	3,54%	7,54	20,20	-5,50	**-0,064**	
	Ar	93,62%	197,97	530,27	92,98%	197,97	530,27	0	0,000	
	O2	1,77%	3,74	10,03	0,84%	1,79	4,80	-5,23	**-0,060**	**0,65**
	N2	0,04%	0,09	0,25	0,14%	0,30	0,80	0,55	0,006	
	CO2	0,02%	0,05	0,14	1,63%	3,50	9,38	9,24	0,101	
**8**	N2O	4,47%	9,63	25,79	4,25%	9,16	24,52	-1,26	**-0,014**	
	Ar	91,84%	197,97	530,27	91,93%	197,97	530,27	0	0,000	
	O2	3,63%	7,83	20,97	2,05%	4,42	11,83	-9,14	**-0,100**	**8,78**
	N2	0,04%	0,09	0,25	1,06%	2,27	6,09	5,84	0,061	
	CO2	0,02%	0,05	0,13	1,69%	3,60	9,65	9,52	0,100	
**9**	N2O	4,53%	9,63	25,80	3,57%	7,63	20,43	-5,37	**-0,056**	
	Ar	93,15%	197,97	530,27	92,61%	197,97	530,27	0	0,000	
	O2	2,25%	4,79	12,83	1,08%	2,30	6,16	-6,66	**-0,070**	**1,07**
	N2	0,05%	0,10	0,27	0,90%	1,94	5,19	4,92	0,050	
	CO2	0,03%	0,06	0,16	1,79%	3,83	10,27	10,11	0,102	
**10**	N2O	4,49%	9,58	25,66	3,59%	7,69	20,59	-5,07	**-0,051**	
	Ar	92,70%	197,97	530,27	92,41%	197,97	530,27	0	0,000	
	O2	2,74%	5,85	15,66	1,31%	2,80	7,49	-8,17	**-0,082**	**1,40**

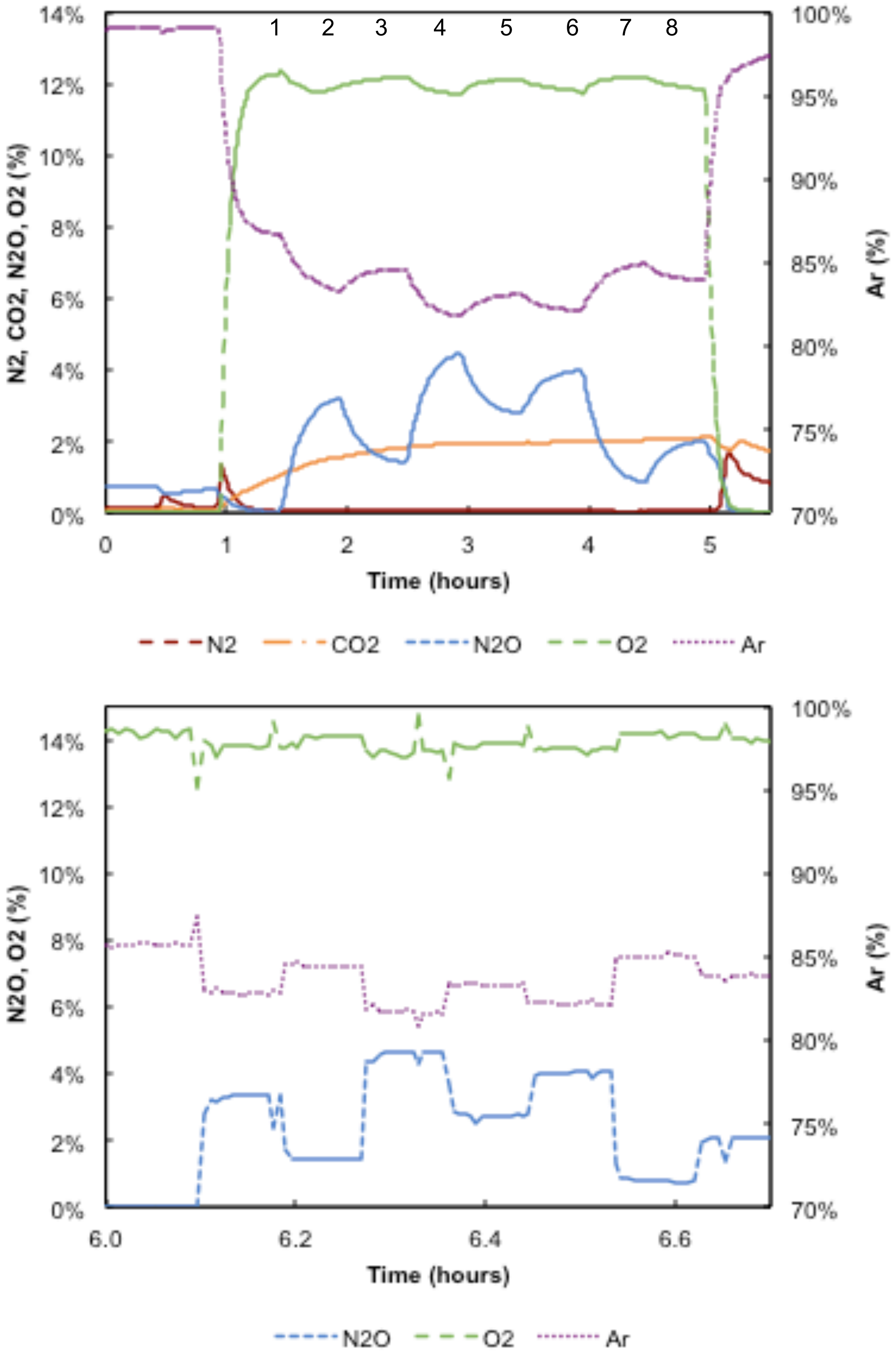

Figure S6 Concentration of N_2O, N_2, CO_2, O_2 and Argon in the offgas (above) and incoming gas (below) of the experiment on day 113 ($O_2 + N_2O$) – **Figure 3c** in the main text. The averaged data for each step is presented in **Table S5.** pH was kept constant at 7.0 ± 0.1

Table S6 Average concentration and rates of N_2O, N_2, CO_2, O_2 and Argon supplied and produced during each of the steps (numbered 1 through 8) in the experiment with simultaneous presence of O_2 and N_2O on day 113 – **Figure 3c** in the main text and **Figure S6**.

		IN-GAS			***OFF-gas***			***R***	***C_L***
			ml/min	*mmol/h*		*ml/min*	*mmol/h*	*mmol/h*	*µM*
	N2	0,02%	0,05	0,13	0,04%	0,09	0,25	0,1	
	CO2	0,01%	0,02	0,06	0,97%	2,21	5,91	5,8	
**1**	N2O	0,02%	0,05	0,12	0,03%	0,06	0,17	0,0	
	Ar	85,93%	197,97	530,27	86,73%	197,97	530,27	0,0	
	O2	14,02%	32,30	86,53	12,24%	27,93	74,82	-11,7	**5,1**
	N2	0,05%	0,11	0,30	0,08%	0,18	0,48	0,2	
	CO2	0,01%	0,03	0,09	1,54%	3,65	9,78	9,7	
**2**	N2O	3,32%	7,94	21,26	3,15%	7,47	20,00	-1,3	
	Ar	82,83%	197,97	530,27	83,38%	197,97	530,27	0	
	O2	13,79%	32,95	88,26	11,86%	28,15	75,40	-12,9	**4,9**
	N2	0,03%	0,07	0,20	0,05%	0,11	0,29	0,1	
	CO2	0,00%	0,00	0,00	1,81%	4,25	11,37	11,4	
**3**	N2O	1,41%	3,31	8,87	1,44%	3,37	9,03	0,2	
	Ar	84,45%	197,97	530,27	84,54%	197,97	530,27	0	
	O2	14,11%	33,07	88,59	12,16%	28,47	76,25	-12,3	**5,1**
	N2	0,06%	0,15	0,40	0,06%	0,19	0,50	0,1	
	CO2	0,01%	0,01	0,04	1,95%	4,72	12,63	12,6	
**4**	N2O	4,61%	11,18	29,95	4,43%	10,71	28,69	-1,3	
	Ar	81,60%	197,97	530,27	81,83%	197,97	530,27	0	
	O2	13,72%	33,29	89,17	89,17%	28,34	75,92	-13,2	**4,9**
	N2	0,04%	0,09	0,25	0,06%	0,14	0,39	0,1	
	CO2	0,04%	0,09	0,24	1,95%	4,65	12,45	12,2	
**5**	N2O	2,72%	6,46	17,29	2,80%	6,67	17,88	0,6	
	Ar	83,33%	197,97	530,27	83,10%	197,97	530,27	0	
	O2	13,88%	32,97	88,31	12,08%	28,78	77,09	-11,2	**5,0**
	N2	0,06%	0,13	0,36	0,07%	0,16	0,43	0,1	
	CO2	0,00%	0,01	0,02	1,99%	4,79	12,82	12,8	
**6**	N2O	4,02%	9,68	25,92	3,97%	9,56	25,60	-0,3	
	Ar	82,21%	197,97	530,27	82,17%	197,97	530,27	0	
	O2	13,71%	33,02	88,45	11,81%	28,45	76,20	-12,3	**4,9**
	N2	0,03%	0,06	0,16	0,04%	0,10	0,27	0,1	
	CO2	0,00%	0,00	0,01	2,03%	4,73	12,66	12,7	
**7**	N2O	0,77%	1,80	4,82	0,89%	2,07	5,54	0,7	
	Ar	85,02%	197,97	530,27	84,87%	197,97	530,27	0	
	O2	14,18%	33,02	88,44	12,17%	28,39	76,03	-12,4	**5,1**
	N2	0,03%	0,08	0,22	0,06%	0,15	0,39	0,2	
	CO2	0,07%	0,16	0,43	2,10%	4,95	13,27	12,8	
**8**	N2O	2,00%	4,71	12,62	2,00%	4,70	12,60	0,0	
	Ar	83,89%	197,97	530,27	83,99%	197,97	530,27	0	
	O2	14,02%	33,08	88,60	11,86%	27,95	74,85	-13,7	**4,9**

Table S7 Parameters and stoichiometry used to calculate the Gibbs free energy dissipation values during microbial growth following the methodology of Kleerebezem and Van Loosdrecht (2010). To obtain the overall stoichiometry of microbial metabolism (MET): first, the stoichiometry of the redox reactions describing the catabolism (**CAT**) and anabolism (**AN**) need to be established from the balanced redox half reactions (**D** for donor half reaction; **A** for acceptor half reaction and **An*** for the anabolic half reaction based on the C and N source – in this case acetate and ammonium). In a subsequent step, the stoichiometric coefficent – here $\boldsymbol{\lambda_{cat}}$, derived from the experimentally determined biomass yields on substrate ($\mathbf{Y_{x/Acetate}}$) - is used to couple the CAT and AN equations into the overall metabolic growth equation. The Gibbs free energy dissipation during the growth reaction is calculated by multiplying the stoichiometric coefficients obtained with the Gibbs free energy of formation of these compounds ($\mathbf{G_f^0}$) corrected for non-ideality considering T = 20 °C and pH 7.

		G_f^0	D	A			An*	CAT			AN	MET		
		kJ mol^{-1}		N_2O	NO_3^-	O_2		N_2O	NO_3^-	O_2		N_2O	NO_3^-	O_2
$\mathbf{Y_{x/Acetate}}$												-0,72	-0,75	-0,89
$\boldsymbol{\lambda_{cat}}$												0,87	0,81	0,59
Ammonium	NH_4^+	-79,4					-0,2				-0,2	-0,2	-0,20	-0,20
Dinitrogen	N_2	0		1	1			4	0,8			3,49	0,65	
Nitrate	NO_3^-	-111,3			-2				-1,6				-1,29	
Nitrous oxide	N_2O	104,2		-1				-4				-3,49		
Carbon dioxide	CO_2	-394,4	2					2	2	2	0,05	1,79	1,67	1,24
Acetate	$C_2H_3O_2^-$	-369,4	-1				-0,5	-1	-1	-1	-0,525	-1,40	-1,33	-1,12
Biomass	$CH_{1.8}O_{0.5}N_{0.2}$	-67,0					1				1	1	1	1
Water	H_2O	-237,2	-2	1	6	2	0,5	2	2,8	2	0,45	2,19	2,71	1,64
Proton (HCl)	H^{+1}	0	7	-2	-12	-4	-0,5	-1	-2,6	-1	-0,325	-1,20	-2,43	-0,92
Oxygen	O_2	0				-1				-2				-1,19
Electron	e^{-1}	0	8	-2	-10	-4	-0,2							
$\mathbf{\Delta G^{01}}$	kJ $CmolX^{-1}$											-1078	-620	-479
$\mathbf{\Delta G^{01}}$	kJ mol N_2O or NO_3^- or O_2											-309	-479	-403
$\mathbf{\Delta G^{01}}$	kJ e^-mol through ETC											-155	-96	-101

References

Conthe M, Wittorf L, Kuenen JG, Kleerebezem R, Hallin S, van Loosdrecht MCM (2018b) Growth yield and selection of nosZ clade II-types in a continuous enrichment culture of N_2O respiring bacteria. Environ Microbiol Rep. doi: 10.1111/1758-2229.126307

Kleerebezem R, Van Loosdrecht MCM (2010) A generalized method for thermodynamic state analysis of environmental systems. Crit. Rev. Environ. Sci. Technol. 40:1–54
